# Immunosenescence in persons with spinal cord injury in relation to urinary tract infections -a cross-sectional study-

**DOI:** 10.1186/s12979-017-0103-6

**Published:** 2017-11-15

**Authors:** David Pavlicek, Jörg Krebs, Simona Capossela, Alessandro Bertolo, Britta Engelhardt, Jürgen Pannek, Jivko Stoyanov

**Affiliations:** 1 Biomedical Laboratories, Swiss Paraplegic Research, Guido A. Zäch Strasse 4, 6207, Nottwil, Switzerland; 2 Swiss Paraplegic Centre, Guido A. Zäch Strasse 1, 6207, Nottwil, Switzerland; 3 Theodor Kocher Institute, University of Bern, Freiestrasse 1, 3012 Bern, Switzerland

**Keywords:** Immunosenescence, Immune frailty, Urinary tract infection, Spinal cord injury, Cytomegalovirus, Memory T-cell

## Abstract

**Background:**

Individuals with a spinal cord injury (SCI), despite specialized rehabilitation and good health care, have a reduced life expectancy. Infectious diseases, such as pneumonias, infected pressure sores and urinary tract infections (UTI) have been identified as the leading causes of mortality. We hypothesise that a premature onset of immune frailty occurs in SCI, possibly caused also by recurrent urinary tract infections.

A cross sectional study was performed comparing blood and urine samples between able bodied controls (*n* = 84) and persons with spinal cord injury (*n* = 85). The results were grouped according to age (below and above 60 years). Assessed were the abundancies of immune cells, the concentration of soluble biomarkers, the in vitro functioning of lymphocytes as well as phenotypic exhaustion of T-cells in blood and urine. Further, the leucocyte telomere length and the cytomegalovirus (CMV) serological status were compared between the groups.

**Results:**

We observed in people with SCI lower proportions of naïve T-cells, more memory T-cells, reduced T-cell proliferation and higher CMV prevalence compared to age-matched controls. SCI participants older than 60 years had a higher prevalence of UTI compared with SCI persons younger than 60 years.

**Conclusion:**

The immune system of people with SCI shows traits of an increased immunological strain and a premature onset of immune frailty. The role of UTI in the onset of immune frailty remains to be elucidated as we did not see significantly higher abundancies of circulating UTI-bacteria specific T-cell clones in persons with SCI. We assume that any impact of UTI on the immune system might be compartmentalized and locally restricted to the urinary tract.

**Electronic supplementary material:**

The online version of this article (10.1186/s12979-017-0103-6) contains supplementary material, which is available to authorized users.

## Background

Persons suffering from a spinal cord injury (SCI) face a broad variety of challenges, SCI linked health issues and biological changes. Even with improved early medical health care, specialized rehabilitation and regular follow up visits today, the longevity of persons with SCI still remains below that of the general population [1, 2]. The leading cause of death are infectious diseases such as pneumonia, infected pressure sores and urinary tract infections (UTI) [3]. The reason for the increased infection rate is still a matter of debate. It has been discussed that the reduced physical activity, the elevated prevalence of depression or the exposure to selected medications in SCI persons have a negative impact on immunological health [4, 5]. There is evidence that the decentralisation of the autologous nervous system causes an acute SCI induced immune deficiency syndrome (SCI-IDS) [6] which manifests as significantly decreased immune cell concentration shortly after a traumatic event. However, a study in humans [7] demonstrated that 4–5 months after the traumatic event, the white blood cell concentration has mostly normalized, but nonetheless infection rates among people with SCI remain high. Monahan et al. [8] could show that individuals with chronic SCI still had reduced CD4 T-cell concentrations but increased numbers of activated (HLA-DR+) CD4 T-cells. Otherwise there is limited data available on the function of the immune system in people with SCI in the chronic phase of the injury and on the precise interplay between cell types and soluble biomarkers of immunity.

Immune frailty (also referred to as immunosenescence), manifests as a decline in immune function, a higher susceptibility to infectious diseases and a reduced response to vaccines [9, 10]. The age related changes affect both the innate- and the adaptive immune system but are most pronounced in the T-cell compartment [11, 12]. Major changes are the phenotypic shift from naïve- to memory T-cells [13] and the decrease in T cell receptor diversity [14]. In addition to the alternations in the composition and diversity of the T-cell pool, the function of the cells is also affected by age. T-lymphocytes from frail persons have typically a reduced in vitro proliferative and signalling response to mitogens compared to their younger counterparts [15]. Paradoxically and in contrast to the reduced function of the immune system a chronic low grade inflammation, termed inflamm–ageing is observed in frail elderly people [16]. Often described are age-dependant elevated basal levels of interleukine-6 (IL-6), C-reactive protein (CRP) and tumour necrosis factor α (TNF-α) [17].

There is growing evidence that a chronic cytomegalovirus (CMV) infection is a major driving force behind immunosenescence [18]. The recurrent stimulation with the CMV antigen leads to several expansion and reduction cycles of the CMV-specific memory T-cells, eventually leading to an accumulation of DNA mutations, shortened telomeres and an exhausted T-cell phenotype [19].

The incidence of SCI peaks in young adults [2] and therefore their immune system is exposed to UTI over the majority of their life. A further complication is that the affected persons often lack sensory function which increases the risk of a prolonged and undetected bacterial infection.

With this study we aim to investigate if there is a premature onset of immunosenescence among persons with SCI which may be partly responsible for the increased infection rates in the chronic phase of a SCI (>1 year after incidence). We further hypothesize, that chronic and recurrent UTI, as they often occur in SCI persons, could have a similar - CMV- like - effect on the immune system. We speculate that due to the increased exposure to UTI causing bacteria the immune system of SCI persons is changed towards a more exhausted and senescent phenotype.

## Methods

### Study design and sampling

A cross-sectional study with a total of 169 participants was performed. Included were men with chronic (since at least one year) SCI and able bodied men who were not on an drug regimen influencing the immune system. Only men were recruited in order to avoid bias through gender specific differences and the inevitable differences in group sizes due to the greater proportion of males among SCI patients. Study participants were divided into four groups: persons with SCI younger than 60 years (ySCI, *n* = 42), persons with SCI aged 60 or older (oSCI, *n* = 43), and as a control, able bodied persons younger than 60 years (yCtr, *n* = 42) and aged 60 or older (oCtr, *n* = 42). After written informed consent had been obtained, venous blood and urine samples were collected during a routine medical check-up.

### Blood processing and differential blood count

Venous blood (27 ml) was collected in 2 × 9 ml K3E S-Monovettes containing EDTA for white blood cell and plasma isolation, 1 × 4 ml K3E S-Monovette containing EDTA for differential blood count and 1 × 4.5 ml S-Monovette for serum isolation (all from Sarstedt). The obtained blood was stored at room temperature and the serum at 4 °C for maximum 5 h after phlebotomy.

Peripheral blood mononuclear cells (PBMC) were isolated by H-Lympholyte Cell Separation Media (Cedarlane, Bioconcept) in a Leucosep tube (Greiner, Sigma-Aldrich) by gradient centrifugation at 800 *g* and room temperature (21 °C air conditioned room) for 20 min. The plasma fraction was collected without disturbing the beneath lying buffy coat and stored in aliquots at −80 °C. In a next step the PBMC containing buffy coat was carefully retrieved and washed with isotonic phosphate buffered saline (PBS), followed by a centrifugation at 210 *g* and room temperature for 10 min. The obtained pellet was resuspended in freezing media - 10% dimethyl sulfoxide, 35% RPMI-1640 medium (Amimed; Bio Concept) and 55% fetal bovine serum (FBS, Amimed; Bio Concept) - adjusted to a concentration of 5*10^6^ cells/ml and immediately transferred to −80 °C in a CoolCell (Biocision) container ensuring a cooling rate of −1 °C/min. The next day, the frozen cell suspension was transferred to a long term cryogenic storage (−150 °C Ultra-low Temperature freezer MDF-C2156VAN, Panasonic).

Simultaneously, a differential white blood cell count and a total serum protein, albumin, C reactive protein (CRP) and creatinine quantification were performed at a certified diagnostic laboratory (at the Swiss Paraplegic Centre in Nottwil, Switzerland).

### Urine processing and analysis

Approximately 30 ml of midstream urine were collected from the controls and the study participants with SCI either using a sterile urine cup and a urine monovette (Sarstedt) or directly from a catheter (intermittent, indwelling or suprapubic). Urine was kept at 4 °C for a maximum of 5 h before being processed. A urine sample was analyzed in a certified clinical laboratory (at the Swiss Paraplegic Centre in Nottwil, Switzerland) for creatinine concentration and tested with a urine-Stix test (Combur 10 Test, Roche) with automated result acquisition (cobas u 4111). In case of a positive urine-Stix signal, a leucocyte and microbial quantification of the urine sediment was performed. If more than 90 leucocytes/ μl or an elevated microorganism count (> 1*10^5^ /ml was detected, a subsequent bacteriological analysis was conducted to identify and characterize the infection causing bacteria. The remaining urine was centrifuged at 4 °C and 1′800 *g* for 10 min. The supernatant was aliquoted and stored at −80 °C until further usage. The urine sediment pellet was resuspended in 1 ml of residual urine and also frozen at −80 °C.

### Immunoglobulins ELISA assays

#### Plasma IgG concentration:

An indirect ELISA was done to measure the concentration of total IgG antibody in plasma as follows: a 96-well plate (Nunc MaxiSorp, Sigma-Aldrich) was coated with 100 μl of 1 ng/μL Protein A (LuBioScience) in PBS and incubated overnight at 4 °C, then washed three times with 100 μl of PBS with a plate washer (Beckman-Coulter, Nyon, Switzerland). Non-specific binding sites were blocked for 2 h at room temperature with blocking solution containing 5% TopBlock (LuBioScience) in PBS. After washing the wells with PBS, thawed plasma aliquots - cleared of cell debris by centrifugation and diluted 20′000 fold in PBS - were incubated for 2 h at room temperature. Supernatants were then removed and the wells washed with PBS. Anti-human-IgG HRP-conjugated secondary antibody (A80-119P, Bethyl) diluted 1:5′000 in blocking buffer was incubated for 1 h at room temperature followed by another washing step with PBS. IgG was determined by colorimetric measurement of the product of the enzymatic reaction mediated by HRP and 100 μl/well of o-phenylenediamine (OPD) solution (15.3 mg/mL in citrate buffer, pH 5.0, Applichem – Axonlab). The reaction was, immediately after the appearance of color (ca. 1–2 min after OPD addition), stopped with 10% sulfuric acid. Absorbance was measured at 450 nm by DTX 880 Multimode Detector (Beckman-Coulter) and IgG concentration (ng/mL) was determined by standard curve made by dilutions of purified human IgG (Bethyl – LuBioScience).

#### In vitro assays:

IgG quantification in the supernatants of stimulated peripheral white blood cells was done with the same ELISA method as described above. Samples were diluted 10 fold in PBS before loading to the plate.

#### Urine IgA concentration:

IgA_total_ concentrations in thawed urine aliquots were assessed by a standard indirect ELISA as follows: a 96-well plate (Nunc MaxiSorp, Sigma-Aldrich) was coated with capture antibody (Goat F(ab’)2 anti-human IgA-UNLAB, Southern Biotech) 1:500 diluted in diluent (0.05% Tween20 + 0.1% bovine serum albumin (BSA) (Albumin fraction V, Applichem Panreac) in PBS). The plate was subsequently incubated overnight at 4 °C, and then washed with 0.05% Tween20 in PBS with a plate washer (Beckman-Coulter). Non-specific binding sites were blocked for 2 h at room temperature with blocking solution containing 1% BSA in PBS. After washing, thawed urine aliquots - cleared of cell debris by centrifugation and diluted 200 fold in diluent - were incubated for 2 h at room temperature. Following another washing step, IgA_total_ (Goat (F(ab’)2 anti-human IgA-biot, Southern Biotech) detection antibodies were added 1:10′000 fold diluted in diluent for 2 h at room temperature. Followed another washing, a 1:8′000 diluted Streptavidin-HRP (Sigma-Aldrich) solution was added to the wells and incubated for 45 min at room temperature. IgA was determined by colorimetric measurement of the product of the enzymatic reaction mediated by HRP and o-phenylenediamine solution and the reaction was stopped with 10% sulfuric acid. Absorbance was measured at 450 nm by DTX 880 Multimode Detector and IgA concentrations (ng/mL) were determined from dilutions of purified human IgA (human IgA kappa-unlab, Southern Biotech).

### Plasma cytokine measurements

The concentrations of cytokines and chemokines were analyzed in the thawed plasma aliquot by Bio-Plex Pro Cytokine, Chemokine and Growth Factor Assay (Bio-Rad Laboratories AG) following the manufacturers protocol: 40 μl of plasma were diluted fourfold in sample dilution buffer. The standard curve concentration was expanded 16-fold in the lower range which still resulted in quantifiable amounts of standards and allowed the measurements of lower cytokine levels. Data were collected and analyzed using a Bio-Rad Bio-Plex 200 instrument equipped with Bio-Plex Manager software (Bio-Rad). We measured the concentrations of interleukin-2 (IL-2), interleukin-4 (IL-4), interleukin-6 (IL-6), interleukin-10 (IL-10), granulocyte-colony stimulating factor (G-CSF), granulocyte-macrophage colony-stimulating factor (GM-CSF), monocyte chemoattractant protein 1 (MCP1) and tumor necrosis factor-alpha (TNF-α).

### CMV serological status

Thawed plasma samples were tested for anti-cytomegalovirus (CMV) IgG with a commercially available ELISA kit (Cusabio Biotech Co.) according to producers manual using 100 μl of plasma. A sample was considered CMV positive when the measured absorption had a value more than 2.1-fold increase compared to the provided negative control.

### T-lymphocyte phenotyping

Peripheral blood lymphocytes (PBL) were thawed in a water bath at 37 °C and immediately diluted 8-fold with PBS containing 10% FBS. The suspension was centrifuged at 210 *g* for 10 min and the supernatant was discarded. After resuspension in 200 μl of PBS flow cytometric analysis of 200′000 cells/ tube was done for the following surface markers: CD3-PerCP (clone SK7, BD biosciences), CD4-APC (clone RPA-T4, BD bioscience), CD8-PE (clone RPA-T8, BD biosciences) and the senescence associated killer cell lectin-like receptor G1 (KLRG1-FITC, clone SA231A2, BioLegend). Cells were incubated with 5 μl of antibodies for 15 min at room temperature, centrifuged at 500 *g* for 5 min and the supernatant was removed. And the cell pellet was resuspended in 200 μl PBS. Cell fluorescence was evaluated with FACScalibur flow cytometer (BD Biosciences) and data were analyzed using FlowJo, 10.0 software (Tree star Inc., USA).

The discrimination of the different memory phenotypes was also done with flow cytometry on freshly thawed cells using the phenotypic subsetting of memory T cells based on the work of Mahnke et al. [20] including the following surface markers: CD4-APC (clone RPA-T4, BD bioscience), CD8-APC (clone RPA-T8, BD bioscience), CD28-FITC (clone CD38.2, BD bioscience), CD45Ro-PE (clone UCHL1, BD bioscience) and CD95-PerCP-Cy5.5 (DX2, BD bioscience). This analysis enables the discrimination between naïve T cells (T_n_: CD4 or CD8^+^, CD45Ro^−^, CD28^+^, CD95^−^), stem cell memory T cells (T_scm_: CD4 or CD8^+^, CD45Ro^−^, CD28^+^, CD95^+^), central memory and transitional memory T cells (T_cm_ + T_tm_: CD4 or CD8^+^, CD45Ro^+^, CD28^+^, CD95^+^), effector memory (T_em_: CD4 or CD8^+^, CD45Ro^+^, CD28^−^, CD95^+^) and terminally differentiated effector memory T cells (T_te_: CD4 or CD8^+^, CD45Ro^−^, CD28^−^, CD95^+^).

### In vitro stimulation of IgG production and quantification

Frozen PBL were thawed in a water bath at 37 °C and then directly diluted 8-fold with PBS + 10% FBS. The suspension was centrifuged at 210 *g* for 10 min, the supernatant was discarded and the pellet resuspended in control medium - RPMI-1640 medium (Amimed; Bio Concept) supplemented with 10% FBS, penicillin–streptomycin (100 units/ml) and amphotericin B (2.5 μg/ml - to reach a concentration of 4 million cells/ml. This cell suspension was kept overnight in a polypropylene Falcon tube allowing gas exchange at humid 37 °C. The next morning a new cell count was performed and 300′000 cells were plated per well of a 96 U-bottom well plate (TTP, Switzerland) in 270 μl of either control (RPMI-1640 + 10% FBS) or stimulating medium, all in duplicates. The used stimulation medium is adapted from a previous work [21] and consisted of 60 ng/ml human recombinant interleukin-2, 25 ng/ml Interleukin-10, 100 ng/ml Interleukin-21 (all from BioBasic Inc.; Stephan Klee Trading and Consulting, Switzerland), the synthetic unmethylated oligodeoxynucleotide deoxycytosine-deoxyguanosine (CpG2429: tcgtcgttttcggcggccgccg, 360 nM [22]; Microsynth AG, Switzerland) and 2.5 μg/ml pokeweed mitogen (Sigma-Aldrich, Switzerland). The cell suspensions were incubated for 7 days at 37 °C in a humid atmosphere and 5% CO_2_ before measuring the IgG concentrations in the supernatants as described above.

### In vitro leucocyte proliferation assay

The thawed PBL, allowed to rest overnight as described above, were used for an in vitro proliferation assay using the fluorescent cell proliferation indicator CytoTell green (AAT Bioquest; LuBioScience), according to the manufactures protocol (20 min incubation at room temperature in 1:600 diluted dye). Cells (150′000 in 270 μl per well) were plated in duplicates in a 96 U-bottom well plate (TTP, Switzerland) either in control medium or in stimulation medium with 2.5 μg/ml pokeweed mitogen or in stimulation medium with 0.1% phytohaemagglutinin (PHA-M, Sigma-Aldrich, Switzerland). The plates were incubated at 37 °C and 5% CO_2_ in humid atmosphere. Proliferative responses of the stimulated PBL and the controls were measured after 7 days of incubation by flow cytometry using the intracellular dye dilution method and following surface marker antibodies: CD3-PerCP (clone SK7, BD biosciences), CD4-APC (clone RPA-T4, BD bioscience), CD8-PE (clone RPA-T8, BD biosciences). Cells were incubated with 5 μl of antibodies for 15 min at room temperature, washed and resuspended in 200 μl PBS. Cell fluorescence was assessed with FACScalibur flow cytometer and data were analyzed using the proliferation tool of the FlowJo, 9.0 software.

### Preparation of a medium containing killed bacteria for in vitro PBL stimulation

Five of the most common urinary tract infection causing bacterial strains from the clinical laboratory at Swiss Paraplegic Centre, (*Klebsiella pneumoniae*, *Proteus vulgaris, Enterococcus sp., Staphylococcus aureus Rosenbach*, ATCC 29213, and *Escherichia coli*) were isolated from urine sediment of SCI-patients. The bacteria were grown overnight in thioglycolate broth at 37 °C. Inactivation was achieved by addition of formaldehyde to a final concentration of 2.5% and an additional overnight incubation at room temperature. Inactivated bacteria were washed three times with PBS to get rid of residual formaldehyde. A spectrophotometric (Diode array spectrophotometer, WPA S2100, Biowave) semi-quantification of the inactivated bacteria was done and the suspensions were normalized to OD_600_ 0.8. The five bacterial strains were pooled together in an equal ratio and diluted 1′000 fold in RPMI-1640 medium supplemented with 10% FBS, penicillin–streptomycin (100 units/ml) and amphotericin B (2.5 μg/ml) for final use.

### Detection of bacteria-specific T-cells

Thawed PBL which were allowed to rest overnight, as described above, were used for the in vitro bacterial stimulation assay. 150′000 cells/well of a 96 U-bottom well plate (TTP, Switzerland) were plated in duplicates in 270 μl of either control medium or medium containing inactivated bacteria (as described above). The plate was incubated at 37 °C and 5% CO_2_ in humid atmosphere. Flow cytometry was used to identify activated T-lymphocytes at day four after plating. Analyzed were the early activation induced surface antigens CD69 and CD137. The measurement of CD69 and CD137 increases the sensitivity and optical discrimination of rare antigen specific T-cells [23, 24]. PBL were stained with the following antibodies: CD4-FITC (clone RPA-T4, BD bioscience), CD8-APC (clone RPA-T8, BD bioscience), CD69-PerCP-Cy5–5 (clone FN50, BD bioscience) and CD137-PE (clone 4B4–1, BioLegend). Cells were incubated with antibodies for 15 min at room temperature, washed and resuspended in PBS. Cell fluorescence was evaluated with FACScalibur flow cytometer and data were analyzed using FlowJo, 10.0 software. Activated cells were characterized as being CD69^+^CD137^+^ and the percentage of bacteria-specific T-cells was calculated by subtracting percentage of the activated unstimulated T-cells from the percentage of the activated bacterial stimulated T-cells.

### Telomere length analysis by PCR

Genomic DNA of freshly thawed PBL was extracted using a commercial kit (Gentra Puregene Cell Kit Qiagen) following manufacturer’s instructions. Telomere length was assessed using a quantitative PCR method as already described elsewhere [25]. Briefly: Standard curves for the telomeres and the reference gene 36B4 were done using synthesized oligonucleotides. The DNA plasmid pBR322 (Sigma-Aldrich, Switzerland) was used to increase the DNA content in the standards reaction mix to match the DNA concentration of the isolated samples. Real-time (RT)-PCR reactions were carried out in duplicates with, 4 ng/μL DNA template, and IQ SYBR Green Supermix (Bio Rad). Specific products were amplified by a quantitative PCR system (CFX96™ Real Time System, Bio Rad). Real-time PCR was carried out with the following settings: denaturation 95 °C, 5 min; followed by 30 amplification cycles of 95 °C, 10 s; 60 °C, 30 s PCR reactions were carried out in a final volume of 20 μL in 96-well PCR plates (Bio Rad). Melting curve analysis was performed after the amplification. Telomere and reference gene 36B4 starting quantities were calculated using the standard curves.

### Statistic tests

A sample size of 42 in each group was determined to be sufficient to detect a one-fold difference in IgA antibody concentration between the groups in the presence of a 100% variance with a power of 90% and a significance level of 5%.

Data are presented as the mean and standard deviation (SD) or the median and the interquartile range (IQR). The data were tested for normal distribution using visual interpretation of QQ-plots and the Kolmogorov-Smirnov test. According to the distribution of the data, parametric or non-parametric tests (Mann-Whitney U test) were used. Statistics were computed using the SPSS Statistics, 24.0 software (IBM, Somers, USA).

## Results

### Demographics, UTI and CMV status of study participants

Table 1 summarizes the demographics and results of blood and urine parameters of the four investigated groups.

**Table 1:** Demographics and prevalence of infections in study participants

	yCtr (±SD)	ySCI (±SD)	oCtr (±SD)	oSCI (±SD)	yCtr-oCtr (*p*-value)	ySCI-oSCI (*p*-value)	yCtr-ySCI (*p*-value)	oCtr-oSCI (*p*-value)
N	42	42	42	43	N/A	N/A	N/A	N/A
Age ± SD	42.8 ± 11	44.1 ± 9	67.6 ± 3	66.9 ± 6	<.001	0.68	<0.001	0.27
Time with SCI (y)	N/A	15.7 ± 11	N/A	21.7 ± 19	N/A	0.26	N/A	N/A
Age at SCI incident	N/A	28.5 ± 11	N/A	44.7 ± 20	N/A	<.001 ↑	N/A	N/A
Para- / Tetraplegic ratio	N/A	1.1	N/A	2.6	N/A	N/A	N/A	N/A
UTI	0%	33%	0%	45%	N/A	N/A ↑	N/A	N/A
CMV+	52%	64%	69%	86%	N/A ↑	N/A ↑	N/A	N/A

Mean values are shown unless otherwise stated

*SD* standard deviation, *N/A* not applicable, *SCI* spinal cord injury, *UTI* urinary tract infections, *CMV+* IgG anti cytomegalovirus present in plasma, **↑ ↓** increasing or decreasing trend with age

A total of 169 volunteers participated in the study and the mean age was similar between the SCI and control groups (Table 1). Individuals of the oSCI group had sustained SCI an average 16.2 years later in life than those of the ySCI group, but the mean time of living with a SCI was similar between the two groups. As expected, no case of UTI, was found in the control groups. The laboratory definition of UTI used in our investigation (> 1*10^5^ bacterial colony forming units/ml urine and >90 leucocytes/μl urine) allows the discrimination from bacteriuria without the assessment of clinical UTI symptoms which are difficult to measure in people with a SCI [26]. The UTI prevalence of persons with SCI aged ≥60 years was about 50% higher compared to their younger counterparts. The prevalence of past and current CMV infections, defined as the presence of circulating anti-CMV IgG antibodies, was greater in the older groups and was substantially higher in the SCI population.

### Blood biochemistry and peripheral blood leucocyte (PBL) telomere length in the study participants

The measurements of a variety of parameters – including immunoglobulins, cytokines and T-cell ratios (Table 2) - revealed that serum total protein levels were significantly lower in the ySCI group compared to the age matched control group. No significant difference was observed in the total plasma IgG levels between the four groups. We measured the plasma levels of six immune related cytokines interleukin (IL-) 2, IL-4, IL-10, granulocyte colony stimulating factor (G-CSF), granulocyte macrophage colony stimulating factor (GM-CSF) and monocyte chemotactic protein 1 (MCP-1) and compared them between the four groups (Table 2).

**Table 2:** Blood biochemistry, CD4/8 cell ratio and PBL telomere length in study participants

	yCtr (IQR)	ySCI (IQR)	oCtr (IQR)	oSCI (IQR)	yCtr-oCtr (*p*-value)	ySCI-oSCI (*p*-value)	yCtr-ySCI (*p*-value)	oCtr-oSCI (*p*-value)
Total prot. (mg/ml)	71.0 (4)	66.5 (7)	70.0 (5)	68.0 (7)	0.06 ↓	0.16 ↔	<.001	0.30
pIgG (mg/ml)	10.7 (4)	9.8 (3)	10.2 (4)	9.7 (4)	0.28 ↔	0.85 ↔	0.30	0.82
IL-2 (pg/ml)	5.5 (3)	6.2 (5)	6.0 (2)	6.1 (2)	0.12 ↔	0.27 ↔	<.01	0.70
IL-4 (pg/ml)	1.5 (0.6)	1.3 (0.2)	2.0 (1.1)	1.4 (0.5)	<.01 ↑	0.14 ↔	<.01	<.001
IL-10 (pg/ml)	3.6 (3)	2.9 (3)	5.0 (4)	3.9 (3)	<.05 ↑	0.12 ↔	0.25	0.19
G-CSF (pg/ml)	26.3 (7)	25.0 (5)	31.0 (10)	26.9 (7)	<.05 ↑	<.05 ↑	<.05	0.05
GM-CSF (pg/ml)	67 (25)	97 (50)	71 (30)	72 (35)	0.14 ↔	<.01 ↓	<.001	0.44
MCP-1 (pg/ml)	138 (28)	131 (20)	153 (43)	134 (27)	<.05 ↑	0.29 ↔	0.10	<.01
CD4/8 (ratio)	2.4 (1.4)	2.1 (2.2)	3.0 (2.7)	2.9 (2.7)	<.05 ↑	0.08 ↑	0.69	0.71
Telomere length PBL (Norm. to yCtr)	1 (0.5)	1.0 (0.4)	0.8 (0.3)	0.9 (0.3)	<.05 ↓	0.09↓	0.44	0.07

Median values are shown

*IQR* inter quartile range is shown in brackets, *N/A* not applicable, *SCI* spinal cord injury, *PBL* peripheral blood leucocyte, ↑ ↓↔ increasing, decreasing or no trend (*p* > 0.1) with age

#### Comparison between the age groups:

Generally, cytokine concentrations were more changing with age in the able bodied group than in the SCI group. The IL-2 plasma levels, a cytokine which is mostly produced by T-cells and promotes their growth, were similar in all four groups. In the controls only, the anti-inflammatory IL-4 and IL-10 were both significantly increased with age. The chemoattractant protein MCP-1 showed a similar pattern: in the controls, it was significantly increased with age, but there was no age effect in the SCI population. Age independent levels of the granulocyte inducing GM-CSF were found in the controls, but the SCI individuals showed a significant reduction in GM-CSF plasma concentration with age. The neutrophil inducing G-CSF was significantly higher in both older groups.

#### Comparison between SCI and controls:

People with SCI had a tendency to lower cytokine concentrations compared with the able bodied controls. IL-2 was significantly higher in the ySCI group compared to the yCtr but there was no statistical difference between oCtr and oSCI. G-CSF and MCP-1 had reduced plasma concentrations in both age categories (younger and older than 60 years). The difference of G-CSF levels was significant between yCtr and ySCI, whereas the difference of MCP-1 was significant between oCtr and oSCI. IL-4 plasma concentrations were significantly lower in ySCI and oSCI groups compared to their age matched able bodied counterparts. No difference between controls and SCI groups was observed in the IL-10 plasma concentrations. GM-CSF was significantly elevated in the ySCI group compared with the yCtr group. However, differences in GM-CSF concentrations between oSCI and oCtr were insignificant.

### PBL telomere length in study participants

Telomere length of peripheral blood mononuclear cells (PBMC) and the CD4/CD8 ratio was measured and compared between the groups (Table 2). Significant differences in telomere length were found between the yCtr and oCtr group. Differences between ySCI and oSCI, or controls and SCI groups were insignificant.

### CD4/CD8 ratio in study participants

A CD4/CD8 ratio below 1 is an immune risk phenotype criterion. Healthy ratio values are reported to be between 1 and 5. We observed that the ratio was increased in the oCtr group compared with yCtr (Table 2). Statistical differences was observed neither between oSCI and ySCI nor between SCI and control groups. In total, only 6 study participants out of 169 had a CD4/CD8 ratio below 1 (2× ySCI, 2× yCtr, 1× oSCI and 1× oCtr).

### Inflamm-ageing in study participants

**Fig. 1:**
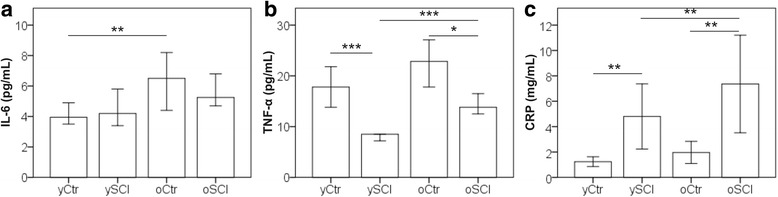
Inflamm-ageing markers among study participants. Inflamm-ageing status was assessed by measuring plasma IL-6 (**a**), plasma TNF-α (**b**) and serum CRP (**c**) levels in the four study groups: able bodied controls <60 years (yCtr, *n* = 42), SCI < 60 years (ySCI, *n* = 41), able bodied controls ≥60 years (oCtr, n = 42) and SCI ≥ 60 years (oSCI, n = 42). Results shown represent the median value for IL-6 and TNF-α and the mean value for CRP. Error bars mark the 95% confidence intervals. Significant differences are indicated as *: *p* < 0.05, **: *p* < 0.01 and *** *p* < 0.001

Figure 1 shows the plasma concentration of three pro inflammatory cytokines IL-6 (Fig. 1a), TNF-α (Fig. 1b) and CRP (Fig. 1c) which were measured to assess the inflamm-ageing status in the study participants. Controls aged 60+ years had on average higher CRP and IL-6 concentrations compared to the young controls, whereas in the SCI groups there was no difference between those two cytokines. In average the oSCI group had significant higher TNF-α levels compared with the ySCI. However the difference in TNF- α concentrations was not significant between the two control groups. The SCI population had, independent of age, higher CRP, but lower TNF-α levels compared with the able bodied control.

### Urine IgA concentrations in study participants

**Fig. 2:**
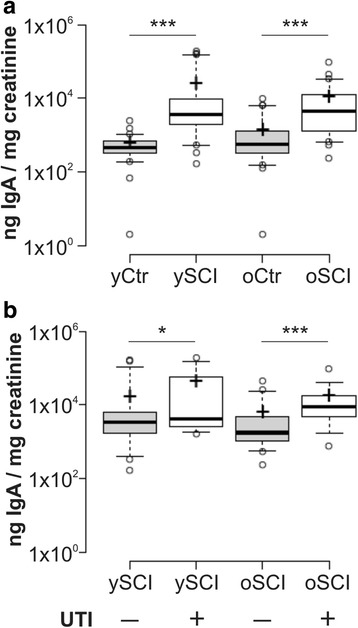
Urine IgA concentrations in study participants. The upper panel (**a**) shows the urine IgA log-values normalized to urine creatinine concentrations for the four groups: able bodied controls <60 years (yCtr, *n* = 42), SCI < 60 years (ySCI, *n* = 42), able bodied controls ≥60 years (oCtr, n = 42) and SCI ≥ 60 years (oSCI, *n* = 43). The bottom panel (**b**) shows the urine IgA log-values normalized to urine creatinine concentrations for the four groups: SCI < 60 years without active urinary tract infection (UTI) (yCtr, UTI-, *n* = 28), SCI < 60 years with an active UTI (ySCI, UTI+, *n* = 14), SCI ≥ 60 years without UTI (oSCI, UTI-, *n* = 24) and SCI ≥ 60 years with UTI (oSCI, UTI+, *n* = 19). Significant differences are indicated as *: *p* < 0.05 and *** *p* < 0.001

The local secretion of IgA in the bladder was measured by detection of IgA in the urine, shown on Fig. 2a. While the mean values of urine IgA concentrations seemed unaffected by age, they were strongly increased in SCI persons, and some participants in the ySCI had very high values (depicted as outliers) compared to the oSCI group. The comparison of urine IgA concentrations in SCI persons with and without an ongoing UTI is depicted in Fig. 2b and shows that persons with an UTI in both age categories have significantly higher IgA levels. While the median difference in the concentration of IgA levels between the young and old SCI groups with an UTI was statistically insignificant, again there was a large shift of mean IgA concentrations with a few very high values observed in the ySCI whereas the oSCI had more homogenous IgA urine levels.

### White blood cell distribution among study participants

**Fig. 3:**
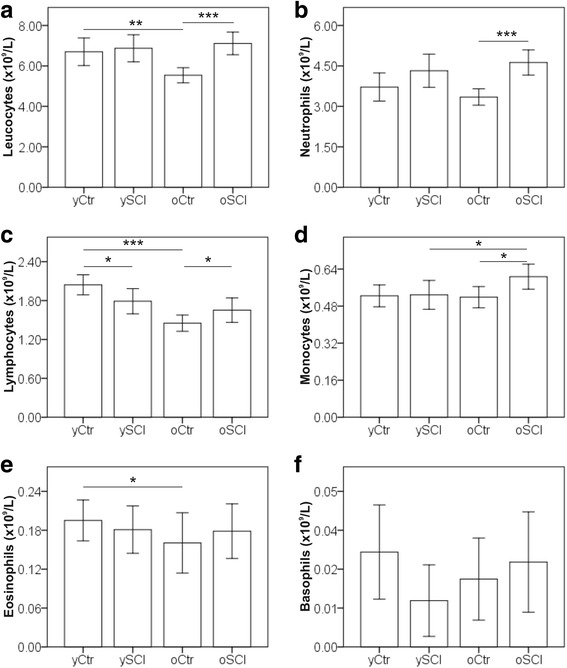
Leucocyte differential counts in study participants. Mean cell concentrations for leucocytes (**a**), neutrophils (**b**), lymphocytes (**c**), monocytes (**d**), eosinophils (**e**) and basophils (**f**) are shown for the four groups: able bodied controls <60 years (yCtr, *n* = 42), SCI < 60 years (ySCI, *n* = 42), able bodied controls ≥60 years (oCtr, *n* = 42) and SCI ≥ 60 years (oSCI, *n* = 43). Error bars mark the 95% confidence intervals. Significant differences are indicated as *: *p* < 0.05, **: *p* < 0.01 and *** *p* < 0.001

The abundancies of total leucocytes, neutrophils, lymphocytes, monocytes, eosinophils and basophils were measured, and the results of the complete white blood cell counts (WBC) are shown in Fig. 3. Total leucocytes (Fig. 3a) concentration did not differ between oSCI and ySCI but was reduced in the oCtr group compared to yCtr. The observed difference in the total white blood cells concentrations can be attributed to the lower concentrations of lymphocytes (Fig. 3c), and eosinophils (Fig. 3e). In the SCI groups only the concentration of the monocytes (Fig. 3d) is different between the two age groups with higher levels in the oSCI cohort. The ySCI group had lower lymphocyte concentrations compared to the yCtr. No difference in cell concentrations of all other leucocyte subtypes was observed between ySCI and yCtr. In the 60+ years groups, the SCI group had significantly higher neutrophil (Fig. 3b), lymphocyte and monocyte counts compared to the controls.

### Abundancy of memory T-cells

In the lymphocytes compartment, we measured T-cells in five different stages of differentiation as shown in Table 3. Flow cytometric analysis of T-cell surface markers such as the co-stimulatory antigen CD28, the Fas receptor CD95, the phosphatase receptor CD45ro and CD4 or CD8 was used to gate on naïve- (Tn), stem cell memory- (Tscm), central memory and transitional memory- (Tcm + Ttm), effector memory- (Tem), and terminally differentiated effector memory T-cells (Tte).

**Table 3:** Subtype abundancies of memory T-cells

		yCtr (SD)	ySCI (SD)	oCtr (SD)	oSCI (SD)	yCtr-oCtr (*p*-value)	ySCI-oSCI (*p*-value)	yCtr-ySCI (*p*-value)	oCtr-oSCI (*p*-value)
	N	41	42	42	41	N/A	N/A	N/A	N/A
CD4	Tn % of CD4	47 (16)	39 (14)	49 (15)	39 (16)	0.66 ↔	0.81 ↔	<0.05	<0.01
	Tscm % of CD4	8.5 (3)	8.5 (3)	9.0 (4)	8.7 (3)	0.71 ↔	0.73 ↔	0.74	0.76
	Tcm + tm % of CD4	42 (14)	50 (13)	39 (14)	48 (17)	0.27 ↔	0.53 ↔	<0.01	<0.01
	Tem % of CD4	2.0 (3)	1.4 (2)	1.9 (3)	2.3 (3)	0.94 ↔	<0.05 ↑	0.57	0.23
	Tte % of CD4	1.0 (1)	0.6 (1)	1.3 (4)	1.3 (2)	0.16 ↔	<0.01 ↑	<0.01	0.32
CD8	Tn % of CD8	32 (18)	34 (17)	19 (12)	14 (13)	<0.001 ↓	<0.001 ↓	0.48	<0.05
	Tscm % of CD8	13 (7)	14 (8)	15 (10)	12 (6)	0.57 ↔	0.52 ↔	0.80	0.44
	Tcm + tm % of CD8	28 (10)	32 (13)	32 (14)	39 (18)	0.21 ↔	0.08 **↑**	0.21	0.12
	Tem % of CD8	8.4 (6)	5.8 (4)	8.1 (6)	9.8 (10)	0.73 ↔	<0.05 ↑	<0.05	0.58
	Tte % of CD8	19 (14)	14 (10)	26 (18)	25 (19)	0.11 ↔	<0.05 ↑	0.19	0.83

Mean values are shown as percentage of total CD4 or CD8 T-cells

*SD* standard deviation is shown in brackets, *N/A* not applicable, *Tn* naïve-, *Tscm* central-, *Tcm + tm* central and transitional-, *Tem* effector-, *Tte* terminally differentiated memory T-cell, ↑ ↓↔ increasing, decreasing or no trend (*p* > 0.1) with age

#### Comparison between the age groups

While there was no significant difference in the CD4 T-cell subtypes between the age groups in able bodied controls, the oSCI group had, compared to ySCI, higher proportions of effector- and terminally- differentiated memory T-cells. CD8 T-cells, seemed to be more affected by age as seen in the decline of naïve cells. Similar findings were observed in the SCI population with the decline of naïve T-cells and the increase in effector- and terminally differentiated memory T-cells.

#### Comparison between SCI and control

The comparison between the control and the SCI group in the CD4 compartment revealed significant differences in the cell memory phenotype abundancies. In both age categories, the SCI group had less naïve- and higher central memory T-cells than the control. In contrast to CD4, the CD8 memory T-cell phenotype distributions were more comparable between the control and SCI groups. As a difference, ySCI individuals had less effector memory CD8 T-cells than the age matched control. However, the oSCI group had less naïve T-cells than the oCtr group.

### In vitro stimulation assays, B-cells, T-cells and UTI burden

**Fig. 4:**
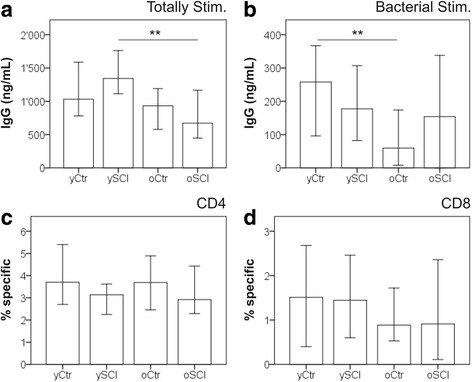
In vitro functional assays of B-cells, T-cells and UTI burden. Median IgG concentrations were measured at day 7 in the supernatants of (**a**) totally stimulated PBL (cultured with IL-2, IL-10, IL-21, pokeweed mitogen and CpG rich DNA sequence) and (**b**) stimulated, with antigens from UTI bacteria (mix of inactivated E.coli, Staph. aureus, Klebsiella pneumonia, Enterococcus spec. And Proteus vulgaris). The bottom panels depict the median abundancies of antigen specific CD4 (**c**) and CD8 (**d**) T-cells as measured as cells with upregulated surface activation markers (CD69 and CD137) after four days of stimulation with UTI bacteria antigens. Participants were split into four groups: able bodied controls <60 years (yCtr, *n* = 41), SCI < 60 years (ySCI, *n* = 42), able bodied controls ≥60 years (oCtr, *n* = 42) and SCI ≥ 60 years (oSCI, *n* = 41). Error bars mark the 95% confidence interval. Significant differences are indicated as **: *p* < 0.01

In vitro stimulation assays allow the assessment of the immune cell function in a controlled setting. Figure 4 summarizes the results of the IgG production potential and the T-cell specificity of in vitro stimulated PBL mixtures. IgG concentrations were measured at day 7 in the supernatants of totally stimulated- (Fig. 4a) and with UTI bacteria antigen stimulated PBL (Fig. 4b). The total stimulation (cocktail of IL-2, IL-10, IL-21, pokeweed mitogen and CpG rich DNA sequence) was considered to be the maximum IgG production capacity. The maximum IgG production capacity was lower in the oSCI group compared with ySCI. No significant difference was observed between oCtr and yCtr. When PBL were specifically stimulated with antigens from UTI bacteria, (mix *of Escherichia coli, Staphylococcus aureus, Klebsiella pneumonia, Enterococcus sp.* and *Proteus vulgaris*), the opposite was observed with significant lower IgG concentrations in the oCtr group compared to the yCtr and no difference in the SCI groups between ySCI and oSCI.

The abundancies of antigen specific CD4 (Fig. 4c) and CD8 (Fig. 4d) T-cells were analysed in PBL stimulated for four days with the UTI bacteria antigen mix. No statistical difference was found between the groups in antigen specific CD4 or CD8 T-cell abundancies.

### In vitro T cell exhaustion and proliferation

**Fig. 5:**
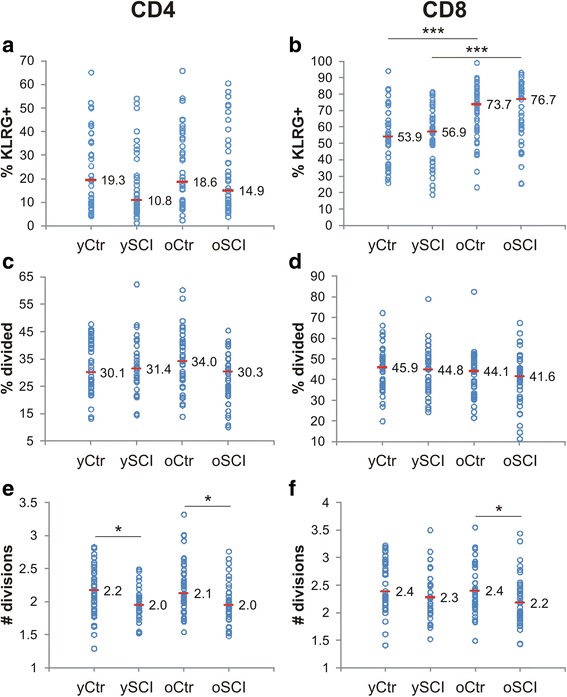
In vitro T-cell exhaustion and proliferation. The top row shows the expression of the surface exhaustion marker “killer cell lectin-like receptor G1” (KLRG) as determined by flow cytometry for CD4 (**a**) and CD8 (**b**) T-cells (*n* = 41, 42, 42 and 41). The bottom four graphs show the results of a 7 days in vitro stimulation of PBL with the mitogen PHA. Middle row depicts the percent of CD4 (**c**) and CD8 (**d**) T-cells which divided at least once or more (*n* = 41, 39, 35 and 39). Bottom row represents the results for the average number of cell divisions for CD4 (**e**) and CD8 (**f**) T-cells after a 7 day in vitro assay with PHA (*n* = 41, 39, 35 and 39). The horizontal mark indicates the median of each group: able bodied controls <60 years (yCtr), SCI < 60 years (ySCI), able bodied controls ≥60 years (oCtr) and SCI ≥ 60 years (oSCI). Significant differences are indicated as *: *p* < 0.05 and *** *p* < 0.001

The results of the phenotypic exhaustion, defined as poor effector function, loss of high proliferative capacity and the sustained expression of inhibitory receptors [27], are shown in Fig. 5. The expression of the co-inhibitory “killer cell lectin-like receptor G1” (KLRG-1) surface marker has been postulated to be a marker of senescence and exhaustion on T-cells [28] and was determined by flow cytometry for the CD4-positive (Fig. 5a) and the CD8-positive (Fig. 5b) T-cells. The results indicate a large inter-individual variance in the KLRG expression for both CD4 and CD8 T-cells. No statistical difference in KLRG expression on CD4 T-cells was found between the four groups. CD8 T-cells seemed to be more affected by age, as both elder groups (oCtr and oSCI) had significant higher proportions of KLRG+ cells compared to the young. No difference in KLRG+ cell ratios was observed between the SCI and the control groups.

The proliferation potential as measured by the in vitro cell division in response to the mitogen phytohaemagglutinin (PHA) was determined with flow cytometry at day 7 by the rate of signal reduction of an intracellular dye (Fig. 5c-f). The number of responsive cells, defined as the percent of cells which divided at least once, observed between the four groups was neither different for the CD4-positive (Fig. 5c) nor for the CD8-positive (Fig. 5d) T-cells. However, cells from SCI study participants divided in average fewer times compared the ones from the controls. The number of divisions for CD4 T-cells (Fig. 5e) was significantly reduced between control and SCI in both age categories whereas in the CD8 T-cell compartment (Fig. 5f) the difference was only significant between the oSCI and oCtr group. On the other hand, age did not have an impact on the number of divisions.

### Impact of SCI duration on immunosenescence parameters

An alternative analysis of data was performed in order to see the impact of SCI duration on the immune frailty parameters. The study participants were grouped according to the duration of SCI in the more recent (≤13.8 years with injury) and long-term (>13.8 years with injury) SCI groups (Additional file 1: Table S1). The threshold of 13.8 years is the median value of SCI duration among participants. Out of 39 compared parameters only 2 differed significantly. People who lived longer with a SCI (>13.8 years) had higher GM-CSF plasma concentrations and lower percentage of CD4 T-cells which responded to an in vitro PHA stimulation assay as described in section 2.9.

## Discussion

The results of the study indicate that the immune system in spinal cord injured (SCI) persons is altered towards a more senescent phenotype, possibly due to a higher antigen exposure of microbial origin - either bacterial or viral. The role of UTI in the onset of premature immunosenescence remains unclear.

Persons with a SCI had on average lower abundancies of naïve- and higher concentrations of memory T-cells when compared with the able bodied control group. This increased shift from naïve to memory phenotype is one of the few accepted hallmarks of immunosenescence [29]. An interesting finding of our study was that the abundancy of naive CD4 T-cells was already reduced in the ySCI compared with yCtr. This suggests, as also others found [4], that the decreased immune function occurs early after an SCI and is maintained thereafter. Memory CD8 T-cell abundancies differed only between oSCI and oCtr which suggests that CD8-T-cells from SCI individuals converted faster from naïve to memory phenotype compared with the control.

T-cell exhaustion, the progressive loss of T-cell effector functions - such as cytokine secretion and the ability for high proliferative capacity - is also known to be caused by some chronic infections [30, 31]. We measured lower responses to the stimulation with PHA in individuals with SCI compared to able bodied controls. The CD4 T-cell responses were already reduced in the ySCI group, whereas the CD8 T-cells differed only between oSCI and oCtr groups. This finding strengthens our hypothesis that the function of CD4 T-cells is affected early after SCI whereas CD8 function changes faster with age. It should be noted, that the number of cells starting division was the same and only the number of divisions was different between SCI and controls.

Unexpectedly, we observed an elevated initial concentration of circulating neutrophils and monocytes in individuals with SCI, simultaneous to the advancing senescence of the adaptive immune system and the increased pathogen susceptibility. With age increasing neutrophilia could be a sign of a higher antigen load or chronic infections [32]. Further indications for an increased antigenic load of the SCI population are manifested in the elevated serum CRP, increased urine IgA, the higher CMV prevalence and the high rate of active UTI. Secretory IgA is known to be induced by the presence of pathogens [33] and is an essential part of the mucosal immunity with protective functions against bacteria in the urinary tract. Generally, a SCI is a risk factor for increased pathogenic antigen exposure at different locations. The common necessity of urethral catheters (indwelling or intermittent) to empty the bladder and the impaired innervation of the bladder facilitate the occurrence of UTI [34]. Artificial breathing devices, immobility, accumulation of liquids in the lungs and the reduced ability of coughing, further increase the risk for respiratory infections [35]. Additionally, due to immobility and the loss of sensation, pressure sores or skin infections arise more likely [36]. Also the long hospital stay following the injury increases the exposure to pathogens. In fact, based on our observations we speculate that the prolonged hospitalisation increases the risk of an CMV infection, which in turn might contribute to the observed immune alterations – a hypothesis which we plan to test in a following project.

However, the question if recurrent UTI are causing a premature onset of immunosenescence still remains unanswered. On one hand, we were able to detect an age conserved stable IgG response to bacterial antigens in people with SCI (whereas the general population has a decreasing IgG response), but on the other hand, we did not observe a higher proportion of specifically responding T-cells in circulation. In contrast to chronic CMV infections which can infect multiple cell- and tissue types [37], UTI remain restricted to the urinary tract. Therefore it is possible that the occurrence of UTI bacteria specific memory T-cells is compartmentalized to the urothelial tissue and surrounding lymph nodes. These tissue resident memory T-cells are found in several body compartments as described elsewhere [38]. Longitudinal studies are necessary to investigate the influence of SCI and recurrent UTI on tissue resident memory T-cells and memory B-cells.

Fortunately, although some changes which point towards an increased immunological strain and senescence were found in persons with SCI, our study could not fully support the hypothesis of a premature onset of immune frailty of the type occurring in octo- and nonagenarians [39]. Typically reported senescence traits as increased inflamm-ageing, shortened PBL telomeres [40] or increased proportions of exhaustions markers (KLRG-1) [41] were similar or even lower in the SCI population compared to the general population.

It is also possible, that the increased medication intake in the SCI group, especially antidepressants and painkillers with anti-inflammatory properties, decreased the plasma concentration of TNF-α [42]. In addition, the observed increase of average PBL telomere length could be a result of the elevated abundancy of innate immune cells in SCI persons as previously seen in other patient populations [43]. Importantly, the higher prevalence of active UTI in the oSCI compared to the ySCI group could be a sign of decreased immune defence in the ageing SCI patients.

Among the limitations of the study are its cross sectional design and the wide range of SCI lesion levels. A major discussion point is if our definition of the groups is suitable to detect age-related changes in the immune system. Given the reduced life expectancy of people with SCI, the old SCI group with individuals ≥60 years was relatively young in order to limit the selective survival bias (Neyman-bias). Despite this precaution, we observed that persons sustaining a SCI early in life were underrepresented in the ≥60 years group. However, the duration of a SCI seems not to affect the immune frailty parameters as observed when analysing our data regarding the time living with a SCI (Additional file 1: Table S1). Out of 39 compared parameters 37 did not differ between more recently injured (≤13.8 years) and longer term injured (>13.8 years) study participants.

Another limitation of the study is that we focused on the adaptive immune system (especially T-cells), while the obtained information on how SCI and aging impacted on the innate immune system or the B-cells beyond antibody production was limited. This gap is planned to be addressed in a following study.

## Conclusions

The immune system of people with SCI shows traits of an increased immunological strain and a premature onset of immune frailty as manifested in higher UTI susceptibility in people above 60 years. Further, persons with SCI had lower proportions of naïve- and increased number of memory T-cells, combined with reduced T-cell proliferation and higher CMV prevalence compared to age matched controls.

The question if recurrent UTI are causing a premature onset of immunosenescence still remains unanswered. We observed an age conserved high IgG response to bacterial antigens but the abundancy of UTI bacteria specific T-cells was comparable between the control and SCI groups.

Data suggest that alterations in the CD4 T-cell compartment, as described above, occur early after a SCI, already in people <60 years, whereas CD8-T cells changes are observed in ≥60 years old individuals.

Further studies - preferably longitudinal - are necessary to investigate signs of immunosenescence in both the adaptive immune system - such as tissue resident memory T-cells, and memory B-cells - as well as in the cells of the innate immune system among the aged SCI persons.

## Additional file

Additional file 1: Table S1. Alternative analysis of data to see the impact of SCI duration on the immune frailty parameters. The study participants were grouped according to the duration of SCI in the more recent (≤13.8 years with injury) and long-term (>13.8 years with injury) groups. Statistical significance was done with a Mann-Whitney U test. (PDF 228 kb)

## Abbreviations

CMV: Cytomegalovirus
CRP: C-reactive protein
PBMC: Peripheral blood mononuclear cells
SCI: Spinal cord injury
SCI-IDS: SCI induced immune deficiency syndrome
UTI: Urinary tract infection
WBC: White blood cells
