# Supplementary material for: Immunosenescence in persons with spinal cord injury in relation to urinary tract infections -a cross-sectional study-
Source: Immun Ageing. 2017 Nov 15;14:22. doi: 10.1186/s12979-017-0103-6 (PMC5688733; doi:10.1186/s12979-017-0103-6)
Supplement: Additional file 1: Table S1. — Alternative analysis of data to see the impact of SCI duration on the immune frailty parameters. The study participants were grouped according to the duration of SCI in the more recent (≤13.8 years with injury) and long-term (>13.8 years with injury) groups. Statistical significance was done with a Mann-Whitney U test. (PDF 228 kb) [file 12979_2017_103_MOESM1_ESM.pdf]

Table S1: Impact of SCI duration

		Recent (≤13.8y) (IQR)	Long-term (>13.8y) (IQR)	p-value
Demographics	N	43	42	N/A
	Age mean ± SD	54.2 ± 16	57.0 ± 11	0.57
	Time (y) with SCI mean ± SD	6.7 ± 4	31.1 ± 13	**<0.001**
	Age at SCI incident mean ± SD	47.3 ± 17	25.8 ± 13	**<0.001**
	Para- / Tetraplegic ratio	2.1	1.3	N/A
	UTI (%)	42	36	N/A
	CMV+ (%)	72	78	N/A
Serum	Total prot. (mg/ml)	68 (8.0)	67 (6.5)	0.40
	CRP mean ± SD (mg/ml)	7.3 ± 13.0	4.9 ± 7.3	0.88
Plasma	IgG (mg/ml)	10.1 (3.8)	9.3 (2.2)	0.11
	IL-2 (pg/ml)	6.0 (3.0)	6.2 (2.6)	0.56
	IL-6 (pg/ml)	4.7 (4.4)	4.7 (4.0)	0.85
	IL-10 (pg/ml)	3.2 (2.8)	3.1 (2.8)	0.48
	G-CSF (pg/ml)	25.6 (5.5)	26.3 (7.3)	0.36
	GM-CSF (pg/ml)	73.0 (37.4)	96.7 (60.2)	**<0.01**
	MCP-1(pg/ml)	132 (19.3)	138 (33.3)	0.11
	TNF-α (pg/ml)	11.2 (9.3)	12.5 (15.9)	0.41
Urine	IgA (µg/mg creatinine)	3.6 (5.9)	4.3 (19.3)	0.19
Cell counts	CD4/8 (ratio)	2.2 (2.5)	2.8 (2.3)	0.35
	Leucocytes ($1*10^9$ /L)	6.4 (1.8)	6.9 (2.4)	0.28
	Neutrophils ($1*10^9$ /L)	4.2 (1.5)	4.2 (2.1)	0.33
	Lymphocytes ($1*10^9$/L)	1.6 (0.8)	1.6 (0.4)	0.76
	Monocytes ($1*10^9$ /L)	0.6 (0.3)	0.6 (0.3)	0.58
	Eosinophils ($1*10^9$ /L)	0.2 (0.2)	0.1 (0.1)	0.91
	Basophils mean ± SD ($1*10^9$ /L)	0.02 ± 0.04	0.03 ± 0.04	0.18

Memory cell subsetting	Tn<br>%of CD4	37.4 (18.8)	38.5 (26.2)	0.95
	Tscm<br>%of CD4	8.4 (3.4)	9.3 (3.8)	0.91
	Tcm+Ttm<br>%of CD4	49.3 (16.1)	49.9 (24.8)	0.75
	Tem<br>%of CD4	1.3 (1.4)	1.0 (1.4)	0.37
	Tte<br>%of CD4	0.2 (1.0)	0.3 (0.4)	0.72
	Tn<br>%of CD8	16.6 (31.7)	18.6 (29.1)	0.31
	Tscm<br>%of CD8	9.9 (8.9)	13.0 (9.9)	0.11
	Tcm+Ttm<br>%of CD8	32.5 (23.7)	35.8 (21.0)	0.41
	Tem<br>%of CD8	6.5 (6)	5.2 (3.4)	0.07
	Tte<br>%of CD8	14.3 (23.7)	15.1 (17.6)	0.75
Senescence markers	Telomere length PBL (Norm. to recent)	1 (1.1)	1.2 (1.4)	0.63
	%KLRG positive CD4	11.6 (19.2)	13.1 (17.3)	0.80
	%KLRG positive CD8	65.7 (33.0)	60.1 (30.2)	0.46
In vitro assays	IgG in supernatant of totally stimulated PBL (ng/ml)	1184 (1337)	1084 (1103)	0.47
	IgG in supernatant of PBL stimulated with UTI bacteria (ng/ml)	236 (472)	80.5 (540)	0.09
	%of bacteria specific CD4	3.3 (3.6)	2.8 (2.2)	0.25
	%of bacteria specific CD8	1.4 (3.6)	1.3 (3.0)	0.77
	%of divided CD4 cells after PHA stimulation	32.4 (10.2)	28.0 (12.5)	**<0.05**
	Number of CD4 divisions after PHA stimulation	2.1 (1.0)	2.4 (1.3)	0.21
	%of divided CD8 cells after PHA stimulation	44.7 (18.4)	40.4 (13.8)	0.09
	Number of CD8 divisions after PHA stimulation	1.9 (0.9)	2.2 (1.1)	0.17

Median values are shown unless otherwise stated. IQR: inter quartile range, SD: standard deviation, N/A: not applicable, Tn: naïve-, Tscm: central-, Tcm+tm: central and transitional-, Tem: effector-, Tte: terminally differentiated memory T-cell, UTI: urinary tract infections.
